# Interferon and anti-TNF therapies differentially modulate amygdala reactivity which predicts associated bidirectional changes in depressive symptoms

**DOI:** 10.1038/s41380-020-0790-9

**Published:** 2020-05-26

**Authors:** Kevin A. Davies, Ella Cooper, Valerie Voon, Jeremy Tibble, Mara Cercignani, Neil A. Harrison

**Affiliations:** 1 Department of Neuroscience, Brighton and Sussex Medical School, University of Sussex Campus, Brighton, BN1 9RY UK; 2 Department of Rheumatology, Brighton & Sussex University Hospitals, Brighton, UK; 3 Department of Psychiatry, University of Cambridge, Cambridge, CB2 0QQ UK; 4 Department of Hepatology, Brighton & Sussex University Hospitals, Brighton, UK; 5 Cardiff University Brain Research Imaging Centre, Cardiff University, Cardiff, CF24 4HQ UK

**Keywords:** Neuroscience, Depression

## Abstract

A third of patients receiving Interferon-α (IFN-α) treatment for Hepatitis-C develop major depressive disorder (MDD). Conversely, anti-Tumor Necrosis Factor (TNF) therapies improve depression providing key empirical support for the “inflammatory theory” of depression. Heightened amygdala reactivity (particularly to negatively valanced stimuli) is a consistent finding within MDD; can predict treatment efficacy and reverses following successful treatment. However, whether IFN-α and anti-TNF enhance/attenuate depressive symptoms through modulation of amygdala emotional reactivity is unknown. Utilizing a prospective study design, we recruited 30 patients (mean 48.0 ± 10.5 years, 21 male) initiating IFN-α treatment for Hepatitis-C and 30 (mean 50.4 ± 15.7 years, 10 male) anti-TNF therapy for inflammatory arthritis. All completed an emotional face-processing task during fMRI and blood sampling before and after their first IFN-α (4-h) or anti-TNF (24-h) injection and follow-up psychiatric assessments for 3 months of treatment. IFN-α significantly increased depression symptoms (Hamilton Depression Rating Scale HAM-D) at 4 weeks (*p* < 0.001) but not 4-h after first dose (*p* > 0.1). Conversely, anti-TNF significantly improved depressive symptoms (Hospital Anxiety and Depression Rating Scale HADS) at both 24-h (*P* = 0.015) and 12 weeks (*p* = 0.018). In support of our a-priori hypothesis, both IFN-α and anti-TNF significantly modulated amygdala reactivity with IFN-α acutely *enhancing* right amygdala responses to sad (compared with neutral) faces (*p* = 0.032) and anti-TNF conversely *decreasing* right amygdala reactivity (across emotional valence) (*p* = 0.033). Furthermore, these changes predicted IFN-induced increases in HAM-D 4 weeks later (*R*^2^ = 0.17, *p* = 0.022) and anti-TNF-associated decreases in HADS at 24-h (*R*^2^ = 0.23, *p* = 0.01) suggesting that actions of systemic inflammation on amygdala emotional reactivity play a mechanistic role in inflammation-associated depressive symptoms.

## Introduction

Inflammation is increasingly implicated in the etiology of depression [1]. Patients with major depressive disorder (MDD) show activated inflammatory pathways including raised pro-inflammatory cytokines [2], acute phase proteins [3], chemokines, and adhesion molecules [4] in blood as well as increased pro-inflammatory cytokines in cerebrospinal fluid (CSF) [5, 6]. Conversely, patients with pro-inflammatory diseases such as rheumatoid arthritis experience a high burden of depressive symptoms, that improves following suppression of peripheral inflammation with cytokine blockers such as the anti-Tumor Necrosis Factor (anti-TNF) therapies [7]. Experimentally induced inflammation can also acutely impair mood and induce anhedonia, effects that have been linked to changes in amygdala, subgenual cingulate, and striatal reactivity to emotional or hedonically valanced stimuli [8–10]. A similar pattern of neuroimaging changes is also observed in MDD [11, 12], suggesting that actions of inflammation on the neural processing of emotionally valanced stimuli may, if prolonged, predispose to the later development of depression.

Studies of patients treated with prolonged courses of pro-inflammatory cytokines e.g., interferon-alpha (IFN-α) provide arguably the most compelling data for an etiological role for inflammation in depression. When used to treat hepatitis-C or malignant melanoma, a third of patients develop major depression, typically within 4–8 weeks of treatment onset [13, 14]. They also provide important mechanistic insights into how actions of systemic inflammation on the brain may precipitate depression. For example, acute (3-h) cortisol and adrenocorticotrophic hormone (ACTH) responses to IFN-α predict the subsequent development of depression implicating the brain’s stress circuitry [15]. Chronic IFN-α also induces the enzyme indoleamine-2,3-dioxygenase to reduce serotonin synthesis and increase metabolically active tryptophan metabolites such as kynurenine [16, 17] and induces circumscribed increases in striatal glucose uptake [18], glutamate concentration [19], and dopamine turnover [20]. However, though cross-sectional, and even a recent prospective study, have repeatedly associated these striatal changes with impairments in motivation and subjective fatigue [21], none has identified a clear association with emergence of depressive symptoms suggesting an alternate neurobiological substrate for these effects.

In contrast, numerous studies have now demonstrated anti-depressant properties of anti-cytokine therapies when used in the context of chronic inflammatory disorders (e.g., inflammatory arthritis) [7, 22] or MDD with raised peripheral inflammatory markers [23]. Preclinical investigations of their mechanistic actions are more limited, though support clinical observations of a rapid (within 24 h) action of anti-TNF therapies on the brain, including importantly, inhibition of amygdala reactivity to emotionally salient (pain) stimuli [24]. This later finding is notable as it highlights a key component of the brain stress-circuitry and complements human studies in MDD where heightened amygdala reactivity to emotionally salient stimuli is a characteristic feature [11, 25]. Furthermore, in MDD, heightened amygdala reactivity has been shown to be highly dynamic, resolving rapidly on successful depression treatment [25] and providing value in predicting therapeutic success [26]. This has led to the proposal that amygdala emotional reactivity may provide the neurobiological substrate for the mood congruent processing bias integral to this disorder [26].

Interestingly, this dynamic nature of amygdala reactivity may underscore why the neural substrates for IFN-α induced depression have remained obscure. To date, studies of IFN-α-induced depression have focused on techniques such as Fluorodeoxyglucose (FDG)-PET that are highly sensitive to resting metabolic activity but not neural reactivity. Consequently, actions on *reactivity* of neural circuits implicated in emotional processing would go un-detected and only become evident using task-based techniques such as fMRI. In support of this, mild inflammatory challenges increase the emotional reactivity [8] but not resting metabolic activity (FDG-PET) [27] of brain regions sub-serving emotional processing including amygdala and subgenual cingulate [8]. Furthermore, though MDD patients typically display heightened reactivity to emotionally valanced stimuli within brain regions sub-serving emotional processing (particularly amygdala) [11, 25] they rarely demonstrate a corresponding change in resting metabolic activity [28].

To address whether acute changes in amygdala emotional reactivity induced by IFN-α/anti-TNF therapies predispose to the later development/resolution of depressive symptoms we recruited 30 patients scheduled to start IFN-α based therapy for chronic hepatitis-C infection and 30 patients scheduled to start anti-TNF based therapy for inflammatory arthritis. All completed an emotional-face processing task known to reliably engage affective brain systems during fMRI on two separate sessions: 1 week before treatment initiation and 4/24 h after their first dose of IFN-α/anti-TNF therapy respectively. All were then followed up for 3 months. We hypothesized that IFN-α would acutely *increase*, and anti-TNF acutely *decrease* amygdala reactivity to emotionally valanced faces. Furthermore, we predicted that if acute changes in emotional reactivity predispose to emergence/resolution of depressive symptoms induced by IFN-α/anti-TNF treatments respectively, these changes should also predict subsequent changes in depressive symptoms.

## Materials and methods

### Participants

Thirty individuals (21 male, mean 48.0 ± 10.5 years) were recruited before initiating IFN-α-based therapy for Hepatitis-C and 30 (10 male, mean 50.4 ± 15.7 years) before initiating anti-TNF therapy for inflammatory arthritis (25 Rheumatoid arthritis, 2 Psoriatic Arthritis, 3 Ankylosing spondylitis). All were fluent in English, aged 18–69 years and fulfilled National Institute for Health and Care Excellence (NICE) guidelines for starting IFN-α-based therapy or anti-TNF therapy. Participants had a baseline psychiatric evaluation of current mental state and previous psychiatric history, using the Mini Neuropsychiatric Inventory. Hepatitis-C participants were excluded if they were receiving treatment for depression at study enrollment, had a history of psychotic or autoimmune illness, substance misuse in the last 6 months, were co-infected with human immunodeficiency virus, or had any cause for liver disease other than hepatitis C. Inflammatory arthritis patients were excluded if they had co-morbid systemic lupus erythematosus, untreated thyroid disease, stroke or multiple sclerosis, or were receiving treatment for depression at study enrollment. The study was approved by Cambridge Central (12/EE/0491) and South East Coast (11/LO/1320) National Research Ethics Committees. All subjects provided written informed consent.

### Study design

The study utilized a prospective cohort design. Participants underwent an emotional face processing task during fMRI, blood draw and behavioral testing at baseline (median 8 days before treatment) and after their first IFN-α (4 h) or anti-TNF (24 h) injection timed to coincide with reported onset of subjective symptoms (but not depressive symptoms per-se) and preclinical data confirming actions of IFN-α and anti-TNF on the brain at these timepoints [15, 21, 24, 29]. Inflammatory response and depressive symptoms were evaluated at each fMRI visit and after 12 weeks of therapy in all participants. IFN-α treated patients underwent additional behavioral assessments at 4 and 8 weeks to capture the more rapid symptom evolution in this group. Consistent with previous studies, profile of mood states (POMS), Hamilton Depression Rating Scale (HAM-D), state and trait anxiety inventory (STAI) and fatigue visual analog scale (fVAS) were used to index changes in mood, depression/anxiety and fatigue respectively in the IFN-α treated group. Arthritis patients underwent a broader assessment designed to capture additional changes in disease activity and pain. This included: POMS, Hospital Anxiety and Depression Scale (HADS), pain and fatigue VAS (pVAS, fVAS), Beck Depression Inventory (BDI), and DAS28, Bath Ankylosing Spondylitis Functional Index and Bath Ankylosing Spondylitis Disease Activity Index or Psoriasis Area and Severity Index and Psoriatic Arthritis Response Criteria as appropriate. They were also given the option to return for a third scanning session at 12 weeks to assess whether any acute changes in emotional reactivity were sustained after 3 months of treatment (24 completed this additional session).

### Emotional face processing task

During each scanning session participants viewed 37 unique positive (happy), 37 negative (sad), and 37 neutrally valanced face stimuli, presented in random order. Each stimulus was displayed for 3 s with a jittered 7-s inter-trial interval. During stimulus display participants were instructed to introspect on how the displayed emotional facial expression reflected their current emotional state. They indicated their response using a button press (not at all/somewhat/quite a lot) immediately after image offset. Two sets of face stimuli were used, counterbalanced across the two imaging sessions. All participants were briefed on the task prior to scanning and all completed the task well.

### fMRI

T2*-weighted echo planar images (EPIs) were acquired on a 1.5 T Siemens Avanto scanner, equipped with a 32-channel phased-array receive-only head coil using a −30° tilted acquisition to reduce orbitofrontal dropout. Each volume provided whole brain coverage (34 interleaved ascending 3 mm slices, 0.6 mm inter-slice gap, echo-time 34 ms: TR 2.52 s). High-resolution T1-weighted anatomical scan was acquired using a magnetization-prepared-rapid-acquisition-gradient-echo sequence to aid group level anatomical localization. EPIs were analyzed in an event related manner using SPM12. Preprocessing consisted of spatial realignment, segmentation, and normalization of the mean EPI image to a standard template then 8 mm^3^ spatial smoothing. The ARTifact Detection Toolbox (http://cibsr.stanford.edu/tools/human-brain-project/artrepair-software.html) [30] was used to identify motion outliers, which were then interpolated in the general linear model. Subject-specific realignment parameters were modeled as covariates of no interest.

We defined left and right amygdala as our primary a priori ROIs based on the prior literature in idiopathic MDD [25, 26], inflammation-associated mood change [8] and depression [31] and preclinical use of anti-TNF [24]. Left and right subgenual cingulate were included as additional ROIs given their reported role in idiopathic depression [32, 33] and inflammation-associated mood change [8]. Masks were produced using the WFU Pickatlas (http://fmri.wfubmc.edu/software/pickatlas).

Stimulus onsets were modeled as 3s events and convolved with a canonical hemodynamic response function. Linear contrasts of regression coefficients were computed at the individual subject level then taken to group level flexible factorial ANOVA or *t*-tests as appropriate. Main effects of viewing emotional faces (happy, sad, neutral compared with implicit baseline) were first tested in each group using paired-sample *t*-tests. Effects of IFN-α and anti-TNF on the processing of all emotional faces (happy, sad, and neutral) and on sad compared with neutral expressions were then tested in flexible factorial ANOVAs (factors: drug (IFN, anti-TNF), time (pre, post drug).

Finally, we performed a regression analysis to investigate whether acute actions of pro (IFN-α) or anti-inflammatory challenges (anti-TNF) on the neural processing of emotionally valanced stimuli predicted the later development/attenuation of depressive symptoms. To minimize variance induced by change in medication (e.g., starting an antidepressant), we restricted this analysis to the next assessment i.e., change in depressive symptoms at 4 weeks in IFN-α group and 12 weeks in the arthritis group. Specifically, we investigated whether acute changes in neural reactivity to sad compared with neutral stimuli or to all emotional expressions within amygdala or subgenual cingulate ROIs predicted the subsequent development of depression symptoms.

### Multiple comparisons

Whole-brain corrected cluster significance was determined using robust Family Wise Error (FWE) correction. Only clusters surviving whole brain or ROI small-volume FWE correction *α* < 0.05 after thresholding at an uncorrected statistical threshold of *p* < 0.001 are reported.

### Behavioral analyses

The primary outcome variable was change in depressive symptoms (HAM-D at 4 weeks in the in the IFN-α group and HADSd at 12 weeks in the anti-TNF group). Data are also reported for changes in each of the other measures. Effects of IFN-α/anti-TNF on depressive symptoms and cytokines were analyzed in SPSS 22.0 using repeated measures ANOVAs and subsequent paired sample *t*-tests. Mauchly’s test of sphericity was performed, and results reported followed Greenhouse-Geisser correction where appropriate. Change in arthritis disease activity is reported for the rheumatoid arthritis patients using the DAS28-CRP = 0.56 × √(TEN28) + 0.28 × √(SW28) + 0.36 × ln(CRP + 1) + 0.014 × GH + 0.96. Where TEN = Tender joint count, SW = Swollen joint count and GH = Patient Global Health assessment from 0 (best) to 100 (worst).

### Cytokine analyses

Blood (20 mL) was drawn into Vacutainer tubes containing ethylenediaminetetraacetic acid (EDTA) anticoagulant, centrifuged at 1300 rpm for 10 min and plasma was removed, aliquoted, and frozen at −80 °C before analysis. Plasma IFN-α was measured using high sensitivity VeriKine^TM^ ELISA (human IFN-α multi-subtype kit, PBL Assay Science, Piscataway, USA). Interleukin-6 (IL-6), Tumor Necrosis factor (TNF), IL-1β and IL-10 using high sensitivity Quantikine^®^ ELISAs (R&D Systems, Abingdon, UK) and IL-1 Receptor antagonist (IL-1Ra) using Quantikine^®^ ELISA. Cytokines were selected based on the prevailing literature to provide an index of both pro- and anti-inflammatory responses.

## Results

### Cytokine response

**Fig. 1:**
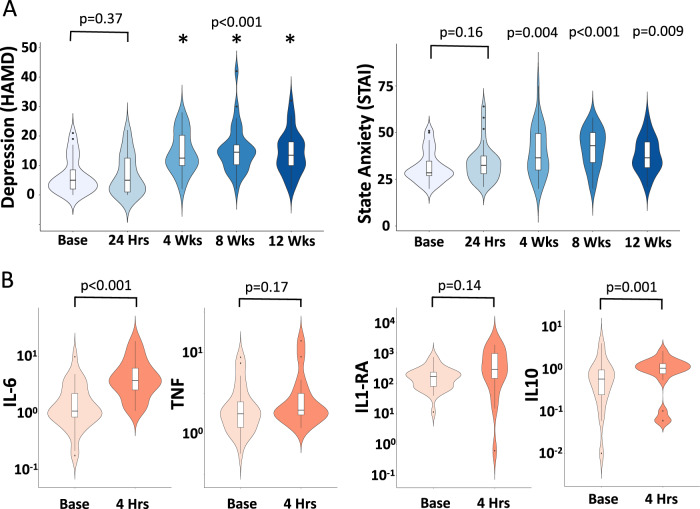
Mood and cytokine responses to interferon-alpha. Violin plot illustrating data distributions (probability density), median and interquartile ranges. **a** Hamilton Depression Rating Scale (HAM-D) and State Anxiety (STAI) at baseline, 4 h, 4, 8, and 12 weeks after starting IFN-α based therapy. *P* values relate to comparison with baseline values. **b** Log plasma Interleukin (IL)-6, Tumor Necrosis factor (TNF), IL1 receptor antagonist (IL1ra) and IL-10 concentration at baseline and 4 h after the first injection of IFN-α.

As anticipated, IFN-α was associated with a significant increase in plasma IFN-α (from mean (± s.e.) 3.12 ± 0.95 pmol/L at baseline to 43.26 ± 7.66 pmol/L at 4 h, *t*_(29)_ = 5.12, *p* < 0.001). Plasma TNF was not significantly altered at this time point (2.51 ± 0.37 to 2.95 ± 0.48 pmol/L, *t*_(29)_ = −0.85, *p* = 0.40. However, we did observe an approximately threefold increase in IL-6 (1.85 ± 0.35 to 4.98 ± 0.69 pmol/L, *t*_(29)_=4.41, *p* < 0.001) as well as significant increases in IL-1 receptor antagonist (IL-1ra) from 191.57 ± 28.52 to 592.20 ± 113.12 pmol/L (*t*_(29)_=3.66, *p* = 0.001) and IL-10 from 0.85 ± 0.18 to 1.40 ± 0.25 pmol/L (*t*_(29)_ = 2.85, *p* = 0.008) demonstrating a broader pro and anti-inflammatory cytokine response to IFN-α (Fig. 1).

**Fig. 2:**
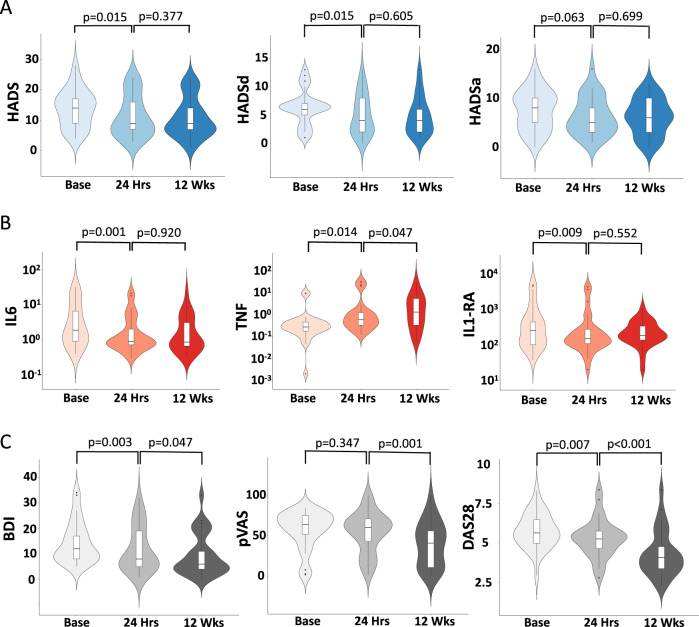
Mood and cytokine responses to anti-TNF therapy. Violin plot illustrating data distributions (probability density), median and interquartile ranges. **a** Hospital Anxiety and Depression Rating Scale (HADS) total and depression (HADSd) and anxiety (HADSa) subscales at baseline, 24 h and 12 weeks after starting anti-TNF therapy. **b** Log plasma Interleukin (IL)-6, Tumor Necrosis factor (TNF) and IL1 receptor antagonist (IL1ra) at baseline, 24 h and 12 weeks after starting anti-TNF therapy. **c** Becks Depression Inventory (BDI), visual analog scale for pain (pVAS) and composite disease activity score (DAS28 CRP) at baseline, 24 h and 12 weeks after starting anti-TNF therapy.

Cytokine responses to anti-TNF (Fig. 2) broadly mirrored those to IFN-α with a halving of IL-6 at 24 h (6.41 ± 1.72 to 3.05 ± 0.97 pmol/L, *t*_(27)_ = −2.55, *p* = 0.017) as well as significant reductions in IL-1ra and IL-10 (766.33 ± 249.22 to 490.74 ± 195.21 pmol/L, *t*_(27)_ = −2.06, *p* = 0.049 and 0.30 ± 0.13 to 0.22 ± 0.12 pmol/L, *t*_(27)_ = −2.53, *p* = 0.017) respectively. TNF concentrations showed a nonsignificant increase at this time point likely reflecting increased circulating bound TNF (0.94 ± 0.45 to 3.19 ± 1.42 pmol/L, *t*_(27)_ = 1.47, *p* = 0.15).

### Depression symptoms

IFN-α significantly increased depressive symptoms (HAM-D: *F*_(4,116)_=29.72, *p* < 0.001) rising from 7.00 ± 1.32 at baseline to peak 16.90 ± 1.75 at 8 weeks, with significant effects observed from 4 weeks until the end of treatment. However, we observed no significant increase in depression symptoms (HAM-D) immediately after IFN-α (7.00 ± 1.32 to 7.50 ± 1.42, *t*_(29)_ = 0.89, *p* = 0.38), nor any association between acute changes in depression (4 h) and subsequent development of depression symptoms at 4, 8, or 12 weeks.

Conversely, anti-TNF significantly reduced depressive symptoms (HADSd) (*F*_(2,56)_ = 4.81, *p* = 0.012) from 6.31 ± 3.07 at baseline to trough 4.82 ± 3.34 at 12 weeks with significant effects observed at both 24 h (*t*_(28)_ = 2.60, *p* = 0.015) and 12 weeks (*t*_(28)_ = 2.51, *p* = 0.018). Similar effects were also observed in cognitive symptoms of depression measured with the BDI (24 h (*t*_(28)_ = 3.25, *p* = 0.003), 12 weeks (*t*_(28)_ = 3.78, *p* = 0.001). HADS anxiety subscale showed a trend toward a reduction in anxiety (24 h: *t*_(28)_ = 1.94, *p* = 0.063; 12 weeks: *t*_(28)_ = 3.25, *p* = 0.053) (Fig. 2). Interestingly, improvement in depressive symptoms at 24 h was not associated with a significant change in joint pain (pVAS t_(28)_ = 0.96, *p* = 0.347). However, there was a significant improvement in the composite measure of disease activity (DAS28-CRP) that includes a subjective rating of global health at this time-point (*t*_(28)_ = 2.92, *p* = 0.007). Clinical and demographic data are summarized in Table 1.

**Table 1:** Demographics of the Hepatitis-C and Inflammatory Arthritis Groups.

	Arthritis (Baseline)	Arthritis (12-Wks)	*p* value	Hep-C (Baseline)	Hep-C (4-Wks)	*p* value
Age (years)	50.4 ± 15.7			48.0 ± 10.5		0.39
Sex						
Male	10			21		0.005
Female	20			9		
Depression Score^a^	6.3 ± 3.1	4.8 ± 3.3	0.018	7.0 ± 7.1	15.6 ± 8.0	<0.001
F-VAS	66.3 ± 20.5	42.0 ± 24.8	<0.001	34.8 ± 26.9	63.4 ± 29.0	<0.001
Additional assessments in the Inflammatory Arthritis group						
P-VAS	57.7 ± 23.5	39.8 ± 27.5	0.001	–	–	–
BDI	14.4 ± 8.1	9.4 ± 8.2	0.001	–	–	–
DAS28-CRP	5.7 ± 1.1	4.3 ± 1.5	<0.001	–	–	–
Additional details for the Inflammatory Arthritis group						
Diagnosis						
Rheumatoid Arthritis (RA)	25					
Psoriatic Arthritis (PsA)	2					
Ankylosing Spondylitis (AS)	3					
Treatment						
Adalimumab	13 (11 RA, 2 PsA)					
Certolizumab	10 (10 RA)					
Etanercept	5 (3 RA, 2 AS)					
Golimumab	2 (1 RA, 1 AS)					

Data represent mean ± standard deviation. F-VAS and P-VAS fatigue- and pain- Visual Analog Scale scores (0–100) respectively; BDI: Beck’s Depression Inventory; DAS28-CRP (28 Item Disease Activity Score).

^a^Values denote HADSd Hospital Anxiety and Depression Rating Scale (Depression component) for Inflammatory Arthritis and HAM-D Hamilton Depression Rating Scale for Hepatitis-C patients.

### Processing of emotionally valanced stimuli

**Fig. 3:**
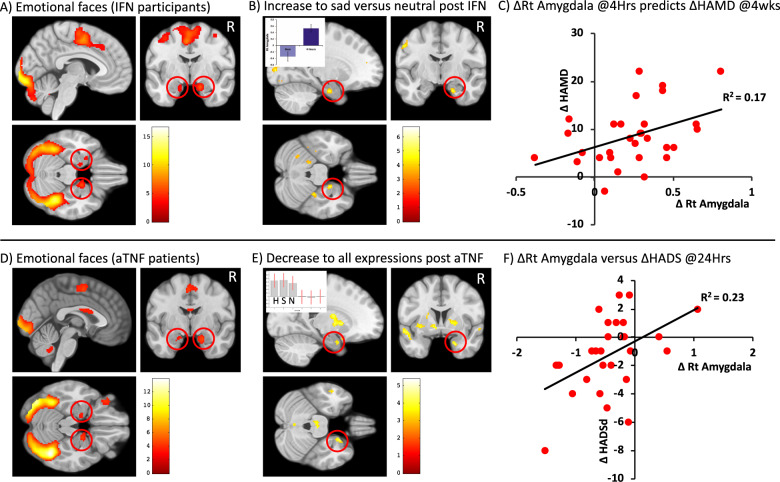
Effects of interferon-alpha and anti-TNF on brain emotional reactivity. Top row Hepatitis-C patients initiating interferon-alpha (IFN). Bottom row inflammatory arthritis patients initiating anti-TNF (aTNF) **a** + **d** Main effect of viewing emotional (sad, happy, neutral) faces versus implicit baseline pre and post IFN and aTNF, respectively. Amygdala responses are indicated with red circles. **b** *Increase* in right amygdala reactivity to sad versus neutral stimuli 4 h after IFN compared with baseline (pre-IFN). Inset shows mean contrast estimate for sad > neutral contrast for the right amygdala ROI at baseline and 4 h after IFN. **c** Correlation between acute change in right Amygdala reactivity to Sad versus neutral face stimuli (4 h minus baseline) and subsequent change in HAM-D score (4 weeks minus baseline). **e** *Decrease* in right amygdala reactivity to ALL emotional stimuli 24 h after IFN compared with baseline (pre-aTNF). Inset shows mean contrast estimate for happy (H), sad (S) and neutral (N) facial expressions for the right amygdala ROI at baseline and 24 h after anti-TNF **f** Correlation between change in right Amygdala reactivity to ALL emotional stimuli (24 h after aTNF minus baseline) and coincident change in HADS depression (HADSd) score (24 h minus baseline). Color scale bars denote *Z* score.

Emotional face stimuli (happy, sad, neutral) in both IFN-α and anti-TNF groups robustly activated the matrix of neural structures encompassing occipital visual cortices, bilateral fusiform face area and bilateral amygdala identified in previous meta-analysis [34] (Fig. 3a, d); and evoked strong activations in bilateral dorsal anterior cingulate and superior parietal lobule and left primary motor cortex confirming task engagement (SI Tables 1 and 2).

We observed no significant main effect of IFN-α (viewing all emotional face stimuli at 4 h > baseline) (Table 2). However in line with our hypothesis, the interaction between IFN-α and processing of negative (sad) compared with neutrally valanced stimuli demonstrated a significant effect within the right amygdala ([18,−6,−28], *Z* = 4.19, FWE small volume correction *p* = 0.032), with sad (compared with neutral) faces inducing a significantly greater right amygdala response 4 h after IFN-α compared with baseline (Fig. 3b, Table 2). Significant interactions were also observed at the whole brain level in bilateral visual cortices and right fusiform face areas confirming a broader impact of IFN-α on negatively valanced emotional processing. Conversely, anti-TNF was associated with a significant *decrease* in right (and left) amygdala reactivity that was observed across all emotional expressions (main effect of IFN) but not significantly different for sad compared with neutral expressions (i.e., no interaction) (Fig. 3e, Table 2). See SI Tables 3–6 for additional results and results at 3 months.

**Table 2:** Effects of Interferon (4 h versus baseline) and anti-TNF (24 h versus baseline) on emotional face processing.

Side	Region	Peak coordinates	*Z* score	*k*	FWE (ROI)
**Interferon: All Emotional Faces (increase)**					
	No significant clusters				
**Interferon: Sad versus Neutral emotional faces (increase)**					
***R***	**1° Visual Cortex**	**[14 −90 4]**	**5.15**	**469**	**<0.001**
***L***	**1° Visual Cortex**	**[−8 −92 18]**	**4.93**	**605**	**<0.001**
***R***	**Fusiform Face Area**	[28–56]	**4.39**	**93**	**0.093**
***R***	**Amygdala**	**[**18–6–28**]**	**4.19**	**33**	**(0.032)**
*L*	Amygdala	[]	n/a	0	n/a
*R*	Subgenual cingulate	[]	n/a	0	n/a
*L*	Subgenual cingulate	[]	n/a	0	n/a
**Anti-TNF: All Emotional Faces (decrease)**					
	**Midbrain**	**[−2 −26 −22]**	**5.14**	**144**	**0.043**
***R***	**Putamen**	**[22 −2 0]**	**5.09**	**413**	**<0.001**
***R***	**Mid temporal**	**[−56 −58 0]**	**4.54**	**182**	**0.016**
***L***	**Superior temporal**	**[−62 −26 10]**	**4.46**	**646**	**<0.001**
***R***	**Anterior cingulate**	**[8 24 26]**	**4.22**	**225**	**0.006**
***R***	**Amygdala**	**[22 2 −28]**	**3.28**	**6**	**(0.033)**
***L***	**Amygdala**	**[−20 −4 −26]**	**3.20**	**3**	**(0.049)**
*R*	Subgenual cingulate	[10 22 −12]	n/a	6	(0.058)
*L*	Subgenual cingulate	n/a	n/a	0	n/a
**Anti-TNF: Sad versus Neutral emotional faces (decrease)**					
	No significant clusters				

Clusters surviving whole brain or region of interest (ROI) (reported in brackets) family wise error (FWE) correction are reported in bold where *k* denotes cluster extent at an uncorrected threshold of *p* < 0.001. Non-significant results for the left amygdala and bilateral subgenual cingulate ROIs are reported in non-bold. [*x* *y* *z*] are MNI coordinates.

To explore how these acute IFN-α/anti-TNF-induced changes in amygdala reactivity contribute to the emergence/resolution of depressive symptoms, we next investigated whether they predicted changes in depressive symptoms at 4/12 weeks, respectively. This regression analysis demonstrated that IFN-α induced changes in amygdala reactivity (sad compared with neutral stimuli) significantly predicted the subsequent development of depressive symptoms at 4 weeks (HAM-D, *F*_(1,29)_ = 5.84, adjusted *R*^2^ = 0.17, *p* = 0.022). Specifically, patients showing the greatest increase in neural reactivity to sad facial expressions after the first dose of IFN-α subsequently showed the greatest increase in HAM-D depressive symptoms (Fig. 3c). Conversely anti-TNF induced changes in amygdala reactivity at 24 h (across emotional expressions) significantly correlated with co-incident changes in depressive symptoms (HADSd, *F*_(1,28)_ = 7.81, adjusted *R*^2^ = 0.23, *p* = 0.01) but not changes in depressive symptoms 12 weeks later (*p* > 0.1) (Fig. 3f). Of note, this significant anti-TNF induced reduction in amygdala reactivity was sustained at 12 weeks (SI Table 3). At this later timepoint, the correlation with coincident change in depressive symptoms persisted, but more weakly and only at trend level in this smaller group (HADSd, *F*_(1,23)_=2.94, adjusted *R*^2^ = 0.12, *p* = 0.10). Results reported were not significantly influenced by sex differences (see SI Results, SI Table 7 and SI Fig. 1).

## Discussion

Here we show that pro- and anti-inflammatory therapies differentially reorient amygdala emotional reactivity. This bidirectional reorientation in amygdala reactivity occurred rapidly (within 4 h of IFN-α injection and 24 h of anti-TNF) and scaled with changes in depressive symptoms. Following IFN-α, this was observed as a selective increase in right amygdala responsivity to negative (sad) versus neutrally valanced expressions and occurred prior to any change in depressive symptoms. Furthermore, it predicted the subsequent development of depressive symptoms, explaining ~17% of the increase in HAM-D symptoms 4 weeks later. In contrast, anti-TNF therapy acutely *decreased* amygdala reactivity across the range of emotional expressions (happy, sad and neutral) with no evidence of any selective effect on sad versus neutral expressions. Again, this change in amygdala reactivity scaled with changes in depressive symptoms (improvement). However, in this group changes in depressive symptoms occurred early (within 24 h) and co-occurred with changes in amygdala reactivity, confirming the association between coincident changes in amygdala reactivity and depressive symptoms but did not provide additional predictive power for further change in depressive symptoms 12 weeks later. Together, these findings extend evidence from experimentally-induced inflammation that link changes in amygdala, subgenual cingulate and striatal emotional reactivity to acute changes in mood [8, 9]. By demonstrating that selective changes in amygdala function *precede* the development of depressive symptoms our findings have relevance beyond IFN-α treatment to suggest a cytokine-mediated mechanism through which sustained systemic inflammation may also predispose to the development of depression.

Within depression, heightened amygdala reactivity to negatively valanced emotional stimuli (sad faces and words) is a reliable finding [25, 28] and has been proposed to mediate the characteristic mood-congruent processing bias [35]. It has value in predicting therapeutic response to CBT [26], normalizes following successful treatment with selective serotonin reuptake inhibitors [35] and in patients in remission serves as a physiological vulnerability marker for relapse [35]. However, the amygdala is also critical to the initiation and integration of broader stress responses [36]. This includes a central role in integrating behavioral and hypothalamic–pituitary–adrenal endocrine responses to extrinsic (e.g., psychological) stressors [36] but also modulation of cortisol stress responses to intrinsic stressors including systemic cytokine challenge [37]. This later finding is noteworthy given earlier evidence that acute ACTH and cortisol responses to Interferon can also predict the later emergence of depressive symptoms [15] and provides a potential mechanistic link between these and our current data.

How IFN-α and anti-TNF act on the brain to alter amygdala reactivity and why they show differential specificity (Increased sensitivity to sad versus neutral expressions, versus decreased sensitivity to all emotional stimuli, respectively) remains incompletely understood, though may be usefully informed by the rodent and non-human primate literature. Firstly, both IFN-α and TNF have been shown to cross the rodent blood-brain barrier (BBB) [38]. Three-fold increases in CSF IFN-α have also been reported from 3 h of systemic IFN-α injection in rhesus monkeys [39] and similar elevations in CSF IFN-α observed after 12 weeks of IFN-α therapy in humans [17]. Further, in rodents, intraperitoneal injection of even modest amounts of IFN-α rapidly induces IFN-sensitive genes within the brain [40, 41] and increases neuronal firing rates within amygdala and other brain regions [29]. Within the hypothalamus, IFN-α can differentially modulate neuronal firing rates in lateral (hunger) and ventro-medial (satiety) related nuclei [29]. Whether, the selective effects of IFN-α on negatively valanced information relates to similar differential effects within the amygdala remains to be determined.

In contrast, raised pro-inflammatory cytokines (particularly TNF and IL-1β) have been reported in CSF in humans and rodent models of Inflammatory Arthritis [42] with recent CSF proteomic analysis showing that anti-TNF therapy reduces CSF concentrations of a range of acute phase and immune response proteins [43]. Interestingly, TNF has also been shown to mediate homeostatic synaptic scaling, the mechanism used by the brain to allow individual neurons to modulate their overall action potential firing rate in response to chronically elevated/reduced activity within their neural circuit [44]. More specifically, TNF increases neuronal post-synaptic AMPA receptor expression resulting in a uniform increase to the strength of ALL synapses to the cell. Theoretically, this mechanism may underlie why we observed a global reduction in amygdala reactivity (across emotional expressions) following anti-TNF therapy.

Use of two complimentary patient groups initiating pro- (IFN-α) and anti- (anti-TNF) inflammatory therapies is a major strength of our study that enabled us to demonstrate that systemic inflammation can rapidly and bidirectionally reorient amygdala reactivity to emotionally valanced stimuli. Further, by following these patients up over time we were able to demonstrate that these acute bi-directional actions on this key component of the brain stress response circuitry scaled with development/resolution of depressive symptoms. However, our use of clinical groups also had a number of inherent weaknesses: For example, we did not enrich the arthritis group for depression at baseline, which may underlie why we observed only a modest reduction in HADSd (~1.5 points or 24%) following anti-TNF. Further, for clinical scheduling reasons we needed to complete this study in a clinical (1.5 T) MRI scanner and to follow up this group at 12 (rather than 4) weeks which may underlie why acute changes in amygdala reactivity predicted only concurrent (but not future) change in depressive symptoms in this group. Nevertheless, our current study extends prior studies implicating acute stress responses in IFN-α-induced depression [15] and provides a potential brain mechanism through which bidirectional changes in peripheral inflammation contribute to the development/resolution of depressive symptoms.

## Supplementary information

Supplemental Information
