# Supplementary material for: Interferon and anti-TNF therapies differentially modulate amygdala reactivity which predicts associated bidirectional changes in depressive symptoms
Source: Mol Psychiatry. 2020 May 26;26(9):5150–60. doi: 10.1038/s41380-020-0790-9 (PMC8589643; doi:10.1038/s41380-020-0790-9)

**SUPPLEMENTARY INFORMATION**

**Interferon and anti-TNF therapies differentially modulate amygdala reactivity which predicts associated bidirectional changes in depressive symptoms**

Kevin A Davies MD^1,2^, Ella Cooper MSc^1^, Valerie Voon MD PhD^3^, Jeremy Tibble MD^4^, Mara Cercignani PhD^1^, Neil A Harrison MD PhD^1,5^

**Supplementary Table 1: Main effect of viewing emotional face stimuli: Interferon participants.**

| **Side** | **Region** | ***Peak voxel*** | ***Z score*** | ***k*** | ***FWE (ROI)*** |
| --- | --- | --- | --- | --- | --- |
| ***R*** | ***Ventral visual stream*** | ***[26 -94 10]*** | ***>8*** | ***12838*** | ***<0.001*** |
| *L* | *Ventral visual stream* | *[-16 -90 -10]* | *>8* |  |  |
| *R* | *Fusiform Face Area* | *[38 -46 -24]* | *>8* |  |  |
| *L* | *Fusiform Face Area* | *[-38 -50 -20]* | *>8* |  |  |
| ***L*** | ***Primary motor cortex*** | ***[-34 -20 48]*** | ***7.29*** | ***4887*** | ***<0.001*** |
| *Bi* | *Dorsal Anterior Cingulate* | *[-4 0 50]* | *7.21* |  |  |
| ***R*** | ***DLPFC*** | ***[58 14 36]*** | ***5.81*** | ***1475*** | ***<0.001*** |
| ***L*** | ***Anterior insula*** | ***[-34 20 0]*** | ***4.80*** | ***445*** | ***<0.001*** |
| ***R*** | ***Anterior insula*** | ***[34 24 -4]*** | ***4.56*** | ***369*** | ***<0.001*** |
| ***R*** | ***Amygdala*** | ***[16 -4 -22]*** | ***5.74*** | ***217*** | ***0.009 (<0.001)*** |
| ***L*** | ***Amygdala*** | ***[-24 2 -28]*** | ***4.20*** | ***113*** | ***(0.003)*** |

Only clusters surviving whole brain or region of interest (ROI) (reported in brackets) family wise error (FWE) correction are reported. k denotes cluster extent at an uncorrected threshold of p < 0.001, [x y z] are MNI coordinates. Bold denotes cluster peak and non-bold sub-cluster peaks. Bi: bilateral, DLPFC: dorsolateral prefrontal cortex

**Supplementary Table 2: Main effect of viewing emotional face stimuli: anti-TNF participants.**

| **Side** | **Region** | ***Peak voxel*** | ***Z score*** | ***k*** | ***FWE (ROI)*** |
| --- | --- | --- | --- | --- | --- |
| ***R*** | ***Ventral visual stream*** | ***[14 -88 -10]*** | ***>8*** | ***11397*** | ***<0.001*** |
| *L* | *Ventral visual stream* | *[-38 -74 -16]* | *>8* |  |  |
| *R* | *Fusiform Face Area* | *[42 -54 -20]* | *>8* |  |  |
| *L* | *Fusiform Face Area* | *[-38 -48 -24]* | *>8* |  |  |
| ***L*** | ***Primary motor cortex*** | ***[-44 -22 58]*** | ***5.12*** | ***441*** | ***<0.001*** |
| ***Bi*** | ***Dorsal Anterior Cingulate*** | ***[-4 -2 54]*** | ***4.71*** | ***268*** | ***0.002*** |
| ***R*** | ***DLPFC*** | ***[58 18 36]*** | ***4.83*** | ***192*** | ***0.012*** |
| *L* | *Anterior insula** | *[-34 16 6]* | *3.80* | *92* | *0.182* |
| ***R*** | ***Anterior insula*** | ***-*** | ***-*** | ***-*** | ***-*** |
| ***R*** | ***Amygdala*** | ***[18 -4 -18]*** | ***4.43*** | ***97*** | ***(0.003)*** |
| ***L*** | ***Amygdala*** | ***[-18 -6 -20]*** | ***3.63*** | ***24*** | ***(0.014)*** |

Clusters surviving whole brain or region of interest (ROI) (reported in brackets) family wise error (FWE) correction are reported. k denotes cluster extent at an uncorrected threshold of p < 0.001, [x y z] are MNI coordinates. Bold denotes cluster peak and non-bold sub-cluster peaks. Bi: bilateral, DLPFC: dorsolateral prefrontal cortex. * region did not survive FWE correction.

**Supplementary Table 3: Brain regions showing reduced emotional reactivity after initiating anti-TNF at 3 months (compared to baseline)**

| **Side** | **Region** | ***Peak coordinates*** | ***Z score*** | ***k*** | ***FWE (ROI)*** |
| --- | --- | --- | --- | --- | --- |
| ***All Emotional Faces*** | | | | | |
| ***R*** | ***Amygdala*** | ***[22 2 24]*** | ***3.33*** | ***6*** | ***(0.033)*** |
| ***L*** | ***Amygdala*** | ***[-22 -6 -22]*** | ***3.86*** | ***26*** | ***(0.01)*** |
| *R* | *Subgenual cingulate* | *n/a* | *n/a* | *0* | *n/a* |
| *L* | *Subgenual cingulate* | *n/a* | *n/a* | *0* | *n/a* |
| ***Sad versus Neutral Emotional Faces*** | | | | | |
|  | *No significant clusters* | | | | |

Clusters surviving whole brain or region of interest (ROI) (reported in brackets) family wise error (FWE) correction are reported in bold where k denotes cluster extent at an uncorrected threshold of p < 0.001. Non-significant results for the left amygdala and bilateral subgenual cingulate ROIs are reported in non-bold. [x y z] are MNI coordinates.

**Supplementary Table 4: Brain regions showing *decreased* emotional reactivity 4 hours after initiating Interferon-alpha based therapy**

| **Side** | **Region** | ***Peak coordinates*** | ***Z score*** | ***k*** | ***FWE (ROI)*** |
| --- | --- | --- | --- | --- | --- |
| ***All Emotional Faces*** | | | | | |
| ***L*** | ***Angular gyrus*** | ***[-48 -68 24]*** | ***5.29*** | ***836*** | ***<0.001*** |
| ***R*** | ***Angular gyrus*** | ***[46 -50 24]*** | ***4.46*** | ***450*** | ***<0.001*** |
| ***L*** | ***Precuneus*** | ***[-12 -58 18]*** | ***4.79*** | ***782*** | ***<0.001*** |
| ***Bi*** | ***Pregenual cingulate*** | ***[2 42 0]*** | ***4.10*** | ***334*** | ***<0.001*** |
| ***Sad versus Neutral Emotional Faces*** | | | | | |
|  | *No significant clusters* | | | | |

**Supplementary Table 5: Brain regions showing *increased* emotional reactivity at 24 hours and 3 months after initiating anti-TNF therapy**

| **Side** | **Region** | ***Peak coordinates*** | ***Z score*** | ***k*** | ***FWE (ROI)*** |
| --- | --- | --- | --- | --- | --- |
| ***All Emotional Faces (increase)*** | | | | | |
|  | *No significant clusters* | | | | |
| ***Sad versus Neutral Emotional Faces (increase)*** | | | | | |
|  | *No significant clusters* | | | | |

**Supplementary Table 6: Effects of anti-TNF on emotional face processing (24hrs versus baseline) for the subset of 25 patients with Rheumatoid Arthritis Only**

| **Side** | **Region** | ***Peak coordinates*** | ***Z score*** | ***k*** | ***FWE (ROI)*** |
| --- | --- | --- | --- | --- | --- |
| ***All Emotional Faces (decrease)*** | | | | | |
|  | ***Midbrain*** | ***[-2 -26 -22]*** | ***4.79*** | ***129*** | ***0.063*** |
| ***R*** | ***Putamen*** | ***[22 -2 2]*** | ***4.56*** | ***353*** | ***<0.001*** |
| ***R*** | ***Mid temporal*** | ***[-56 -58 0]*** | ***4.48*** | ***174*** | ***0.019*** |
| ***L*** | ***Superior temporal*** | ***[-64 -24 8]*** | ***4.57*** | ***312*** | ***0.001*** |
| ***R*** | ***Anterior cingulate*** | ***[10 24 26]*** | ***4.58*** | ***193*** | ***0.012*** |
| ***R*** | ***Amygdala*** | ***[20 0 -28]*** | ***3.18*** | ***4*** | ***(0.043)*** |
| *L* | *Amygdala* | *[-12 -4 -24]* | *3.10* | *1* | *(n.s.)* |
| *R* | *Subgenual cingulate* | *[10 20 -12]* | *3.23* | *5* | *(0.063)* |
| *L* | *Subgenual cingulate* | *n/a* | *n/a* | *0* | *n/a* |
| ***Sad versus Neutral emotional faces (decrease)*** | | | | | |
|  | *No significant clusters* | | | | |

**Notes:** This Rheumatoid Arthritis subset of 25 patients had a similar age to the whole inflammatory Arthritis group: Mean 53.4 (±15.7 ) years compared to the whole group: mean 50.4 (±15.7) years. They also had a similar mean HADSd depression score at baseline: 6.0 (versus 6.31 for the whole group) and showed a similar reduction in HADSd scores after 12 weeks of anti-TNF therapy: 4.38 (versus 4.82 for the whole group). Paired sample t-test in HADSd scores 12 weeks versus baseline p=0.016 (versus p=0.018 for the whole group).

**Supplementary Results**

**Assessment of potential impact of sex differences**

The gender distribution of the two study groups was significantly different reflecting the sex-specific prevalence of Hepatitis-C and Inflammatory Arthritis (Table 1). To further explore any potential impact of this on our results we compared data across groups using mixed measures ANOVAs (between subject factor: treatment (IFN-α, anti-TNF), within-subject factor: time (pre, post drug)) with and without sex included as a covariate of no-interest or additional between-subject factor. In addition, we explored effects of sex within each group (IFN-α, anti-TNF).

**A. Cytokines:**

**Summary:** Inclusion of sex as a co-variate of no-interest or additional between-subject factor did not significantly alter reported cytokine responses to treatments. Within each group, there were no significant sex differences in cytokine responses to treatment.

**IL6**: Treatment x Time interaction F(1,58)=36.9, P<0.001 remained significant after inclusion of sex as a co-variate F(1,57)=34.0, P<0.001 and there were no significant interactions with Sex when included as an additional between subject factor p>0.1. No significant interaction with sex within either group (p>0.1)

**TNF:** Treatment x Time interaction F(1,58)=4.9, P=0.03 remained significant after inclusion of sex as a co-variate F(1,57)=4.1, P=0.047 and there were no significant interactions with Sex when included as an additional between subject factor p>0.1. No significant interaction with sex within either group (p>0.1)

**IL1ra:** Treatment x Time interaction F(1,58)=6.6, P=0.013 remained significant after inclusion of sex as a co-variate F(1,57)=5.7, P=0.020 and there was no significant interaction with Sex when included as an additional between subject factor p>0.1. No significant interaction with sex within either group (p>0.1)

**IL10:** Treatment x Time interaction F(1,58)=9.4, P=0.003 remained significant after inclusion of sex as a co-variate F(1,57)=12.1, P=0.001 and there was no significant interactions with Sex when included as an additional between subject factor p>0.1. No significant interaction with sex within either group (p>0.1)

**B. Depressive symptoms:**

Inclusion of sex as a co-variate did not significantly change reported effects of IFN-α or anti-TNF therapy on depressive symptoms either acutely or at later timepoints. i.e. after controlling for sex, acute effects of IFN-α (4 hour) on depressive symptoms remained non-significant (p>0.1) and effects at all later timepoints remained significant at the reported threshold (p<0.001). After controlling for sex, effects of anti-TNF on depressive symptoms were marginally stronger (At 24 hours: p=0.004 versus p=0.015; 12 weeks: p=0.001 versus p=0.018).

Inclusion of sex as an additional factor did not reveal significant interactions with sex on acute changes in depressive symptoms. However, it did reveal a significant Group x Time x Sex interaction (F(1,57) = 8.7, p=0.005) on late (12 week) responses to treatment, with women showing significantly greater increases in depressive symptoms following IFN-α than men (F(1,28)=6.4, p=0.018) and significantly better improvement in depressive symptoms following anti-TNF (F(1,28)=4.9, p=0.035) (see SI Figure 1). In view of this, we also repeated our regression analyses of acute changes in amygdala reactivity and later changes in depressive symptoms as reported in C below.

**C. Neuroimaging:**

Inclusion of sex as a regressor of no-interest did not significantly change effects of IFN-α and anti-TNF on the processing of all emotional faces (happy, sad and neutral) or sad compared to neutral expressions tested in flexible factorial ANOVAs (factors: drug (IFN, anti-TNF), time (pre, post drug) with F tests revealing significant effects in each of the reported regions including amygdala at the same statistical thresholds. To investigate this further, we included sex as a covariate of no-interest in t-tests performed to investigate effects of pro- and anti-inflammatory therapies separately in each group. Again, this did not significantly alter our reported findings (SI Table 7).

The relationship between anti-TNF induced changes in right amygdala reactivity and depressive symptoms remained significant after removing variance associated with Sex (adjusted R^2^=0.21, p =0.016) as did the relationship between IFN-α induced changes in right amygdala reactivity and depressive symptoms 4 week later (adjusted R^2^=0.18, p =0.021)

**Supplementary Table 7: Effects of Interferon (4 hours versus baseline) and anti-TNF (24hrs versus baseline) on emotional face processing after correction for sex**

| **Side** | **Region** | ***Peak coordinates*** | ***Z score*** | ***k*** | ***FWE (ROI)*** |
| --- | --- | --- | --- | --- | --- |
| ***Interferon: All Emotional Faces (increase)*** | | | | | |
|  | *No significant clusters* | | | | |
| ***Interferon: Sad versus Neutral emotional faces (increase)*** | | | | | |
| ***R*** | ***1° Visual Cortex*** | ***[14 -90 4]*** | ***5.06*** | ***463*** | ***<0.001*** |
| ***L*** | ***1° Visual Cortex*** | ***[-8 -92 18]*** | ***4.87*** | ***591*** | ***<0.001*** |
| ***R*** | ***Fusiform Face Area*** | ***[28 -56 -12]*** | ***4.33*** | ***85*** | ***0.093*** |
| ***R*** | ***Amygdala*** | ***[18 -6 -28]*** | ***4.22*** | ***33*** | ***(0.032)*** |
| *L* | *Amygdala* | *[]* | *n/a* | *0* | *n/a* |
| *R* | *Subgenual cingulate* | *[]* | *n/a* | *0* | *n/a* |
| *L* | *Subgenual cingulate* | *[]* | *n/a* | *0* | *n/a* |
| ***Anti-TNF: All Emotional Faces (decrease)*** | | | | | |
| ***R*** | ***Putamen*** | ***[22 -2 0]*** | ***5.02*** | ***417*** | ***<0.001*** |
| ***R*** | ***Mid temporal*** | ***[-56 -58 0]*** | ***4.41*** | ***148*** | ***0.038*** |
| ***L*** | ***Superior temporal*** | ***[-62 -26 10]*** | ***4.24*** | ***352*** | ***<0.001*** |
| ***R*** | ***Anterior cingulate*** | ***[8 24 26]*** | ***4.44*** | ***237*** | ***0.004*** |
| ***R*** | ***Amygdala*** | ***[18 2 -24]*** | ***3.38*** | ***7*** | ***(0.033)*** |
| ***L*** | ***Amygdala*** | ***[-20 -4 -26]*** | ***3.20*** | ***3*** | ***(0.049)*** |
| ***R*** | ***Subgenual cingulate*** | ***[10 22 -12]*** | ***3.51*** | ***9*** | ***(0.047)*** |
| *L* | *Subgenual cingulate* | *n/a* | *n/a* | *0* | *n/a* |
| ***Anti-TNF: Sad versus Neutral emotional faces (decrease)*** | | | | | |
|  | *No significant clusters* | | | | |

Clusters surviving whole brain or region of interest (ROI) (reported in brackets) family wise error (FWE) correction are reported in bold where k denotes cluster extent at an uncorrected threshold of p < 0.001. Non-significant results for the left amygdala and bilateral subgenual cingulate ROIs are reported in non-bold. [x y z] are MNI coordinates.

**Supplementary Figure 1:** Sex-specific changes in depressive symptoms following A) Interferon-alpha and B) anti-TNF therapies

**A**


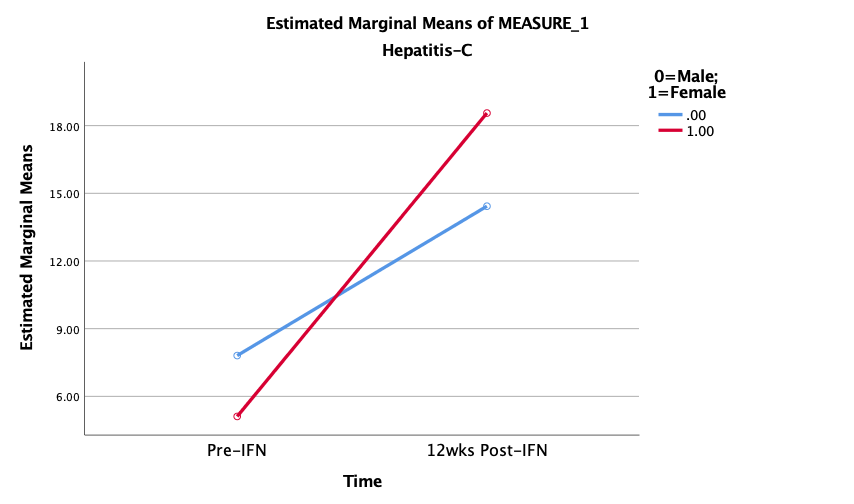


**B**


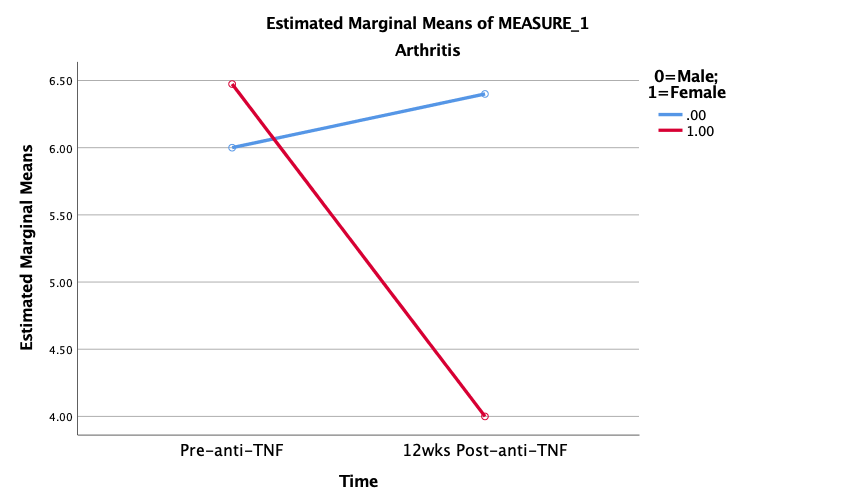

Supplement: Supplementary file 1 — Supplemental Information [file 41380_2020_790_MOESM1_ESM.docx]
